# Differences in muscle activity and temporal step parameters between Lokomat guided walking and treadmill walking in post-stroke hemiparetic patients and healthy walkers

**DOI:** 10.1186/s12984-017-0244-z

**Published:** 2017-04-20

**Authors:** Klaske van Kammen, Anne M. Boonstra, Lucas H. V. van der Woude, Heleen A. Reinders-Messelink, Rob den Otter

**Affiliations:** 1 University of Groningen, University Medical Center Groningen, Center for Human Movement Sciences, P.O. Box 196 21, A. Deusinglaan 1, 9713 AV Groningen, The Netherlands; 2 Rehabilitation Center ‘Revalidatie Friesland’, Beetsterzwaag, The Netherlands; 3 University of Groningen, University Medical Center Groningen, Center for Rehabilitation, Groningen, The Netherlands

**Keywords:** Stroke, Electromyography, Robotics, Neurorehabilitation, Gait, Lokomat

## Abstract

**Background:**

The Lokomat is a robotic exoskeleton that can be used to train gait function in hemiparetic stroke. To purposefully employ the Lokomat for training, it is important to understand (1) how Lokomat guided walking affects muscle activity following stroke and how these effects differ between patients and healthy walkers, (2) how abnormalities in the muscle activity of patients are modulated through Lokomat guided gait, and (3) how temporal step characteristics of patients were modulated during Lokomat guided walking.

**Methods:**

Ten hemiparetic stroke patients (>3 months post-stroke) and ten healthy age-matched controls walked on the treadmill and in the Lokomat (guidance force 50%, no bodyweight support) at matched speeds (0.56 m/s). Electromyography was used to record the activity of Gluteus Medius, Biceps Femoris, Vastus Lateralis, Medial Gastrocnemius and Tibialis Anterior, bilaterally in patients and of the dominant leg in healthy walkers. Pressure sensors placed in the footwear were used to determine relative durations of the first double support and the single support phases.

**Results:**

Overall, Lokomat guided walking was associated with a general lowering of muscle activity compared to treadmill walking, in patients as well as healthy walkers. The nature of these effects differed between groups for specific muscles, in that reductions in patients were larger if muscles were overly active during treadmill walking (unaffected Biceps Femoris and Gluteus Medius, affected Biceps Femoris and Vastus Lateralis), and smaller if activity was already abnormally low (affected Medial Gastrocnemius). Also, Lokomat guided walking was associated with a decrease in asymmetry in the relative duration of the single support phase.

**Conclusions:**

In stroke patients, Lokomat guided walking results in a general reduction of muscle activity, that affects epochs of overactivity and epochs of reduced activity in a similar fashion. These findings should be taken into account when considering the clinical potential of the Lokomat training environment in stroke, and may inform further developments in the design of robotic gait trainers.

**Electronic supplementary material:**

The online version of this article (doi:10.1186/s12984-017-0244-z) contains supplementary material, which is available to authorized users.

## Background

A cerebral vascular accident is one of the most common causes of walking disabilities, with approximately 60% of the patients suffering from persistent problems in walking [1]. These impairments are associated with decreased walking speed and stride length [2], spatial and temporal asymmetry [2–5], and a higher fall risk [6]. As walking represents a key aspect of independent functioning, regaining safe gait function is one of the main goals in stroke rehabilitation. Robot assisted gait training (RAGT) is a relatively novel approach to gait training, with robotic devices such as the Lokomat (Hocoma AG, Volketswil, Switzerland) now commercially available. The Lokomat is an actuated exoskeleton that guides the limbs through the gait cycle, making it possible to elicit a kinematically normal gait pattern in patients who are incapable of independent stepping [7]. In order to purposefully use the Lokomat for gait rehabilitation, knowledge is needed on how guided walking in the Lokomat affects the neuromuscular control that underlies hemiparetic gait.

Locomotor training that involves a high number of task specific movement repetitions is generally associated with larger increases in functional gait performance [8]. In principle, highly intensive gait exercise can be implemented in over-ground or (bodyweight supported) treadmill training, by letting the therapist manually support limb movements. However, such a training approach is strenuous and physically demanding for therapists [9–12], in particular when training patients with a low ambulatory status. By mechanically guiding the limbs through the gait cycle, robotic gait trainers such as the Lokomat provide a less demanding training setting for the therapist, thus allowing patients to make many repetitions of a well-defined, normative gait pattern [12]. The amount of support (or ‘guidance’) provided by the exoskeleton can be set to the requirements of the patient and the training, but is kept constant throughout the whole gait cycle. Arguably, the experience of successful, normative and symmetric stepping induces task-specific sensory information that may guide locomotor control and inform plastic changes in the central nervous system [9, 11, 12]. However, offering robotic guidance may also reduce the need for active contribution by the patient, which is an important prerequisite for activity-dependent learning [13–15]. Understanding the potential of the Lokomat for gait training therefore requires knowledge on the extent to which the walker actively contributes to exoskeleton guided gait. To monitor levels of active involvement, studying muscle activity through electromyography (EMG) may be particularly useful. Research on healthy gait suggests that Lokomat guided walking is associated with reduced muscular output compared to regular treadmill walking [16], and that the amplitude of muscle activity is negatively associated with the amount of guidance that is provided [17]. Although these findings suggest that guided exoskeleton walking reduces the need for the active control of limb movements, knowledge is lacking on whether the same holds true for patient groups that are targeted for Lokomat training, such as stroke patients..

It is well established that post-stroke hemiparetic gait is altered due to insufficient supraspinal drive, spasticity and peripheral changes in the muscle [3, 18]. At the neuromuscular level, these aberrations are expressed as abnormal muscular amplitudes and a disrupted temporal ordering of muscle activity, in both the affected and unaffected limb [3, 19–22]. In addition, hemiparetic gait is often characterized by temporal and spatial asymmetry of the stepping pattern [2–5], as patients tend to avoid standing on their affected leg. These abnormalities may require different control strategies in response to the robotic guidance provided by the Lokomat exoskeleton, than previously observed in healthy subjects. However, little is known about the muscle activity that underlies hemiparetic walking in the Lokomat. Only a recent study by Coenen and coworkers [23] showed that during Lokomat guided walking, muscle activation patterns of stroke patients were more symmetrical, and more similar to healthy treadmill walking. It must be noted that this study involved patients with relatively good walking abilities that were capable of independent overground walking, whereas robot assisted gait training may be specifically indicated for patients with a more severely impaired ambulatory function (see [7] for a review). Furthermore, no experimental control was exerted over body weight support (BWS) and the levels of exoskeleton guidance, as they were set individually for each patient. Whilst these parameters are known to affect muscle activity [17, 24–26].

The present study wished to elaborate on this work, and assess the difference in muscle activity and temporal control of stepping between Lokomat guided gait and treadmill walking under controlled BWS and Lokomat guidance conditions, in patients with more severe walking problems. More specifically, the aim of this study was threefold. First, we wished to determine how Lokomat guided walking affects muscle activity following stroke and how these effects differ between patients and healthy walkers. To this end, the gait-related muscle activity during Lokomat guided walking and unrestrained treadmill walking were compared between hemiparetic walkers and a group of healthy peers. Second, we aimed to establish how abnormalities in the muscle activity of patients are modulated through Lokomat guided gait. We therefore identified abnormalities during treadmill walking by comparing patients and healthy walkers, allowing us to address how these aberrations are modulated in the Lokomat. Finally, we wanted to determine how temporal step characteristics of patients were modulated during Lokomat guided walking.

## Methods

### Participants

Ten chronic stroke patients (8 females, 64.4 ± 6.3 years) and ten gender and age-matched healthy controls (7 females, 62.7 ± 4.8 years) volunteered to participate. Patients had a first ever unilateral stroke (infarction or haemorrhage), were at least 3 months post stroke, had unilateral paresis of the leg, and a Functional Ambulation Classification [27] score of at least 2 (*in our study operationalized as: ‘patient needs continuous or intermittent support of one person to help with balance or coordination’*) and at the most 4 (*in our study operationalized as: ‘patient can walk independently in and around the house (<200 m) with help of walking aids, on level ground, but requires help when walking > 200 m, on stairs, slopes and uneven surfaces*’). Patients were excluded when they had severely impaired cognitive functions (Mini Mental State Exam [28] score ≤ 25), severe speech, language or communication disorders, severe visual problems or neglect, or co-morbidities that are known to affect gait or balance performance. Stroke patients were excluded when they were incapable to walk under experimental conditions. None of the healthy subjects suffered from disorders that are known to affect gait performance or muscle activity. None of the participants had previous experience with walking in the Lokomat. For a full overview of the characteristics of the participants, see Table 1.

**Table 1:** Overview of participant characteristics

Subject	Gender	Age (years)	Weight (kg)	Length (m)	Affected Side (L/R)^a^	Type stroke	Post stroke (months)	FAC^b^	Walking aids
Stroke patients									
1	Female	57	66	1.76	L	Hemorrhage	5	2	Wheelchair, AFO^c^
2	Female	72	68	1.69	R	Hemorrhage	70	4	Cane, AFO^c^
3	Female	70	70	1.63	L	Infarction	12	3	Cane
4	Female	72	68	1.68	R	Infarction	216	4	Cane
5	Female	67	50	1.68	R	Infarction	23	4	Cane, one-handed rolling walker, AFO^c^
6	Female	55	86	1.70	L	Hemorrhage	53	3	Eifel cane, wheelchair
7	Male	57	72	1.76	R	Hemorrhage	5	4	Cane, adjusted shoes
8	Female	65	65	1.59	L	Hemorrhage	148	4	Rolling walker
9	Male	66	91	1.83	L	Infarction	27	4	Cane, AFO^c^
10	Female	63	60	1.57	L	Infarction	4	3	Cane, wheelchair, AFO^c^
*Mean (std)*		64.4 (6.3)	69.9 11.8)	1.69 (0.08)			56,3 (71,5)		
Healthy subjects									
*Mean (std)*	7 females	62.7(4.8)	77.1(13.0)	1.74(0.09)					

^a^ *Left/Right;* ^b^ *Functional Ambulation Clasification Score;* ^c^ *Ankle Foot Orthoses*

All participants provided their written informed consent. The protocol was in accordance with the Declaration of Helsinki [29], and approved by the Medical Ethical Committee of the University Medical Center Groningen (METc UMCG, project number: NL46137.042.12), the Netherlands.

### Experimental protocol

For this study, the Lokomat Pro version 6.0 (Hocoma AG, Volketswil, Switzerland) was used, which is a bilaterally driven exoskeleton that is combined with a BWS system and a treadmill (for more detailed information see ‘apparatus’ section). The Lokomat was located at the rehabilitation centre ‘Revalidatie Friesland’ in Beetsterzwaag, the Netherlands. Stroke patients visited the rehabilitation centre twice. The first session was not used for testing but only to familiarize patients with the Lokomat, and to evaluate whether they were capable to walk under the experimental walking conditions. The data collection was conducted in the second session (i.e. the test session). Healthy subjects visited the rehabilitation centre only once, for the test session.

During testing the protocol involved two walking conditions, (i) on the Lokomat treadmill, but disengaged from the exoskeleton (henceforth ‘treadmill walking’) and (ii) on the Lokomat treadmill attached to the exoskeleton, that provided 50% guidance (henceforth ‘Lokomat guided walking’). Since muscle activity can be affected by gait speed [30, 31] and BWS [24–26], these parameters were kept constant throughout the experiment by setting gait speed to 0.56 m/s and providing no BWS for any of the participants. Settings for gait speed and the level of guidance were chosen based on earlier research [23] and on clinical experience of physiotherapist working with stroke patients in our centre.

To avoid that muscle activity during treadmill walking was confounded by after-effects of Lokomat guided walking, treadmill trails were always conducted before walking in the Lokomat, for all participants. To minimize possible effects of carry-over between walking conditions, and to eliminate possible effects of fatigue, a resting period of at least 5 min was obligatory between the treadmill and Lokomat trial. Patients were allowed extra resting time when needed, until they indicated to be ready for the Lokomat trial. Trial durations were 60 s, and when the Lokomat suddenly stopped, e.g. when unexpected movements triggered the safety mechanism, the trial was repeated until a trial of the required duration was completed. Prior to a trial, participants were allowed practice time, until they indicated to be comfortable with the specific settings. Participants were allowed to rest their hands on the side bars of the Lokomat for stability and wore the BWS harness for safety only, without providing BWS. During Lokomat guided walking ankle movements were stabilised by elastic foot lifters. Patients wore their own (adjusted) footwear and Ankle Foot Orthoses.

### Apparatus

#### The Lokomat Pro

The Lokomat exoskeleton is comprised of two actuated orthoses that are attached to the participant’s limbs by means of cuffs and straps. The geometry (hip width, length of the upper and lower limbs) of the orthoses, and the size and position of leg cuffs were adjusted to the subject’s individual anthropometry, ensuring that walking in the device was as natural and comfortable as possible [9].

The hip and knee joints of the Lokomat are actuated by linear drives that move the orthoses through the gait cycle, in the sagittal plane [9, 10, 12], and as such ‘*guide*’ the participant’s limbs to move along a predefined path. This predefined pattern is based on joint movements derived from trajectories of healthy walkers [9, 10], but can be fine-tuned by adjusting the hip- and knee angles to meet walkers functionality. Ankle movements are not actuated, but can be stabilized by elastic foot lifters, to prevent foot drop and concomitant stumbling during the swing phase.

The ‘*guidance*’ provided by the Lokomat exoskeleton used in the present study is realized by means of an impedance controller, that allows the level of guidance to be set by the therapist. The level of guidance that is offered determines how much limb movements are permitted to deviate from a predefined pattern. As long as the patient moves along the predefined pattern, the controller does not interfere, but once the limits are exceeded, joint torques are applied to move the limb back towards the desired trajectory [32]. When guidance is set to its maximum (i.e. 100%), the walker is forced to strictly follow the predefined pattern, but when guidance is set to nil, free limb movements are allowed as exoskeleton torques are applied only to correct for the exoskeleton inertia [10, 12, 17].

In the present study, the level of guidance during the ‘Lokomat guided walking’ condition was set to 50%, which allows small deviations and requires more active involvement of the walker compared to fully guided walking.

#### Electromyography and detection of gait events

To assess muscle activity, surface EMG was used to measure activity of the Gluteus Medius (GM), Vastus Lateralis (VL), Biceps Femoris (BF), Medial Gastrocnemius (MG) and Tibialis Anterior (TA). Since stroke patients are known to display abnormal neuromuscular control in both the affected and unaffected limb [3, 19–22], EMG was measured bilaterally in patients. However, in healthy participants only EMG of the dominant leg was measured, as Ôupuu and Winter [33] showed that in a group of healthy walkers EMG does not differ between the dominant and non-dominant leg. Signals were recorded using self-adhesive, disposable Ag/AgCl electrodes (Kendall/Tyco ARBO; Warren, MI, USA) with a 10 mm diameter and a minimum electrode distance of 25 mm. Sensor placement conformed to the SENIAM guidelines [34]. To improve skin conduction, the electrode sites were prepared by removing body hair, and by abrading and cleaning the skin with alcohol.

Custom-made insoles equipped with 4 pressure sensors (FSR402, diameter 18 mm, loading 10 – 1000 g, one under the heel and three under the forefoot) were used to detect initial foot contact and swing onset for both legs. Pressure sensor and EMG signals were simultaneously sampled at 2048 Hz and fed to a Porti7 portable recording system (Twente Medical Systems, Enschede, The Netherlands). The unit (common mode rejection of >90 dB, a 2μVpp noise level and an input impedance >1 GV) pre-amplified and A/D converted (22 bits) the signals before storage on a computer for offline analysis.

### Data analysis

#### Signal analysis

Offline analysis of pressure sensor and EMG data was done using costum-made software routines in Matlab (version 2015b; The Mathworks Inc., Natick, MA). In line with SENIAM recommendations [35], EMG data were firstly high-pass filtered using a 4^th^ order Butterworth with a cut-off frequency of 10 Hz, to reduce movement artefacts. Subsequently, the data were full wave rectified and low-pass filtered (10 Hz 4^th^ order Butterworth). Pressure sensor data were used to distinguish the first double support (DS1), the single support (SS), the second double support (DS2) and the swing (SW) phase, for both legs. The summed (rectified and low-pass filtered) EMG data was calculated for each of these sub-phases and subsequently averaged over all strides, for each participant and each condition, for further statistical processing. For visual presentation of the data only, the filtered EMG data of each individual step were time-normalized with respect to gait cycle time (i.e. 0 – 100%, heelstrike to heelstrike), and subsequently averaged over strides. To assess the temporal structure of the hemiparetic gait pattern, relative durations (expressed as a percentage of the total gait cycle time) of the DS1 and SS phase were calculated and averaged over strides for each of the limbs, of stroke patients only. Similar to earlier patient-related EMG studies [19, 23, 36], no amplitude normalization was performed on the EMG data. Normalization of EMG amplitude reduces the inter-individual variation in EMG amplitude by dividing the measured amplitude in microvolts by e.g. the maximum amplitude measured over all conditions, so that EMG values are expressed as a percentage of the maximal amplitude. A potential limitation of this procedure is that, if the signal to noise ratio is low (e.g. when the overall amplitude of the signal is low, which is not uncommon following stroke [37, 38]), background noise will have a disproportional contribution to the amplitude normalized signal, which may result in unreliable group averages. Therefore, it was chosen not to normalize the EMG amplitude.

#### Statistical analysis

To compare levels of muscle activity between treadmill walking and Lokomat guided walking (within subjects factor ‘Condition’), and to determine whether the effects of walking condition differed between stroke patients and healthy walkers (between subjects factor ‘Group’), a repeated measures ANOVA was conducted separately for each of 4 sub-phases (DS1, SS, DS2, and SW).

To elucidate how aberrant reactions of patients to Lokomat guided walking were related to abnormalities as displayed during unrestrained treadmill walking, a supplementary analysis was conducted. In case of a significant Condition by Group interaction (implying that differences between Lokomat guided walking and treadmill walking were dissimilar for patients and healthy walkers), independent sample t-tests were conducted to compare the groups during treadmill walking. This way, abnormalities in the EMG of patients were determined for unrestrained treadmill walking, allowing us to specifically address how these abnormalities (i.e. abnormally high or abnormally low activity) were modulated during Lokomat guided walking.

To determine whether temporal step asymmetries in patients were modulated during Lokomat guided walking, a Repeated Measurements ANOVA was conducted on the duration of the DS1 and SS phase, to compare the effects of the within subject factors ‘Limb’ (affected vs unaffected leg) and ‘Condition’ (treadmill walking vs. Lokomat guided walking). We specifically focused in the Limb by Condition interactions, indicating that temporal asymmetry in patients was altered through Lokomat guided walking.

Statistical analyses were performed with SPSS version 20 for Windows (SPSS, Chicago, IL,USA). All test results were evaluated with an alpha of 5%, and the Benjamini-Hochberg correction was used to correct for multiple testing [39]. All analyses were done separately for each gait phase.

## Results

### Muscle activity

**Fig. 1:**
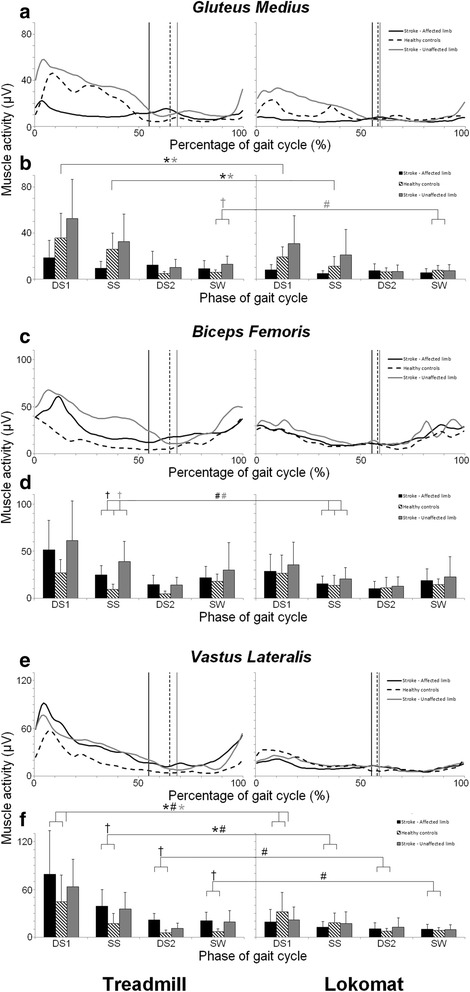
EMG profiles and average muscle activity per gait phase for **(a, b)** Gluteus Medius, **(c, d)** Biceps Femoris and **(e, f)** Vastus Lateralis. **a, c, e** *:* Time normalized EMG profiles (μV) during treadmill walking (left panel) and Lokomat guided walking (right panel), for the affected limb (black lines) and the unaffected limb (grey lines) of stroke patients, and the dominant limb (black dashed line) of healthy walkers. The vertical lines indicate stance-swing transition for the affected limb (black lines) and the unaffected limb (grey lines) of stroke patients, and the dominant limb (black dashed line) of healthy walkers. **b,d,f** *:* Average level of muscle activity and standard deviations (μV) during treadmill walking (left panel) and Lokomat guided walking (right panel), for the affected limb (black bars) and the unaffected limb (grey bars) of stroke patients, and the dominant limb (black dashed bars) of healthy walkers, for four subphases of the gait cycle (DS1: first double support phase; SS: single support phase; DS2: second double support phase; SW: swing phase). Statistical results are indicated for affected limb of stroke patients vs. limb of healthy subjects (black signs) and unaffected limb of stroke patients vs limb of healthy subjects (grey signs): * significant main effect of Condition (ANOVA), ^#^ significant Condition by Group interaction (ANOVA), † significant effect of Group during treadmill walking (independent *t*-test)

**Fig. 2:**
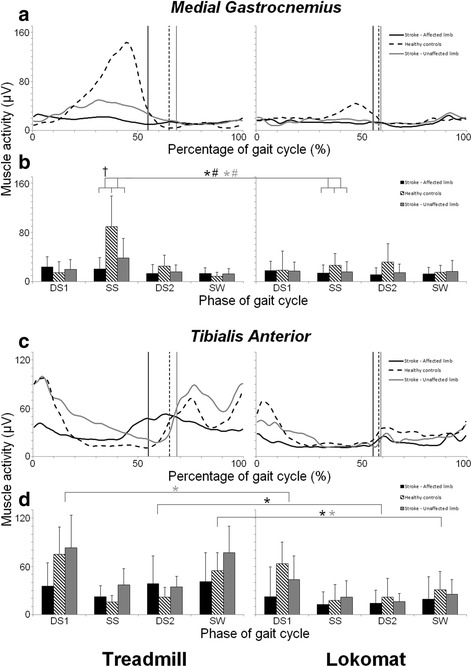
EMG profiles and average muscle activity per gait phase for **(a, b)** Gastrocnemius Medialis and **(c, d)** Tibialis Anterior. **a, c** *:* Time normalized EMG profiles (μV) and **b, d** *:* Average level of muscle activity and standard deviations (μV). See Fig. 1 for further details

The ensemble averaged EMG profiles, and the mean EMG values (+ standard deviation (SD)) for each of the four sub-phases, are shown in Fig. 1 (GM, BF, VL) and Fig. 2 (MG, TA).

The Repeated Measures ANOVA’s revealed significant main effects of ‘Condition’ in all but the BF muscle, indicating that a general lowering of muscular amplitude occurred during Lokomat guided walking for both stroke patients and healthy walkers, when compared to treadmill walking, (see Fig. 1a + c and Fig. 2 a + b).

The Repeated Measures ANOVA’s additionally revealed a number of significant Condition by Group interactions, indicating that the magnitude of the decrement was different for stroke patients and healthy subjects. A supplementary set of t-tests allowed to assess these differences in more detail, revealing that overall the detected group related effects of Lokomat guided walking, were strongly related to the abnormalities displayed by patients during treadmill walking. More specifically, for affected BF^SS^, VL^SS,DS2,SW^ and unaffected GM^SW^, BF^SS^ activity was abnormally high during treadmill walking, compared to healthy walkers (see Fig. 1a-c). For the majority of these muscles and phases (with exception of VL^DS1^), larger Lokomat-induced reductions in activity were found in patients, compared to healthy walkers. For the MG^SS^ of the affected leg the opposite was true, i.e. the Lokomat-induced reductions in activity were larger in healthy walkers compared to patients. The supplementary analysis revealed that this was the result of the already abnormally low MG^SS^ activity in the affected limb of stroke patients during treadmill walking, compared to healthy walkers (see Fig. 2a).

The combined results of the main analysis and supplementary analysis of the EMG data indicate that group-related differences between walking conditions (treadmill vs. Lokomat) were apparent, and that most of these differences were linked to a reduction of group differences (i.e. abnormally high or abnormally low activity in the stroke group) displayed during treadmill walking. Activity during Lokomat guided walking was generally low, and similar between both groups. For a complete overview of the statistical results for the EMG data, the reader can consult Additional file 1: Tables S1 and S2.

### Step phase durations

**Fig. 3:**
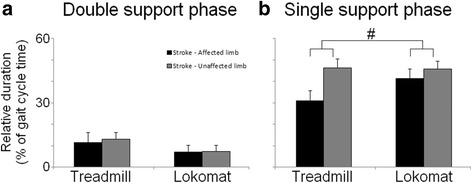
Mean duration of step phases. The mean relative duration (+ standard deviations) of **a** *:* the double support phase and **b** *:* the single support phase during treadmill walking and Lokomat guided walking, for the affected limb (black bars) and the unaffected limb (grey bars) of stroke patient, expressed as a percentage of the total gait cycle duration. Statistical results are indicated: ^#^ significant Condition by Limb interaction (ANOVA)

For the stroke group, a Repeated Measures ANOVA was conducted to assess if and how temporal step asymmetries were affected by Lokomat guided walking. Figure 3 shows the mean (+SD) relative durations of DS1 and SS phase. A significant Limb by Condition effect for the SS phase indicated that Lokomat guided walking reduced temporal asymmetry. More specifically, whereas during treadmill walking the relative duration of the SS phase was longer in the unaffected limb compared to the affected limb, these durations were similar in the Lokomat. For a complete overview of the statistical results for the step phase duration data, Additional file 1: Table S3.

## Discussion

The aims of this study were to assess how Lokomat guided walking affects muscle activity following stroke and how these effects differ between patients and healthy walkers, (2) how abnormalities in the muscle activity of patients are modulated through Lokomat guided gait, and (3) how temporal step characteristics of patients were modulated during Lokomat guided walking. The results show that in both groups, Lokomat guided walking was associated with lower levels of muscle activity. In patients, these reductions were observed irrespective of whether activity was abnormally high or abnormally low during treadmill walking. The temporal step asymmetry that patients displayed during treadmill walking was reduced on the Lokomat. These findings provide valuable information on the active muscular contributions of hemiparetic stroke patients to the production of Lokomat guided gait.

### Lokomat guided gait is characterized by a general reduction in muscle activity

Active neuromuscular control is required to accommodate locomotor task demands (e.g. support, balance, propulsion, and foot clearance). If patients are incapable of independently producing the necessary control signals (e.g. because of muscle weakness), the provision of robotic guidance may help to successfully produce stepping, by assisting leg movements and simplifying support and balance demands. Offering guidance may result in an overall reduction in the active contribution [40–42], which was also clearly reflected in present results. Compared to treadmill walking, Lokomat guided walking was associated with a reduction in muscle activity in both stroke patients and healthy walkers, confirming earlier findings [16, 17, 23, 40].

A supplementary analysis allowed us to specifically address if and how Lokomat guided walking affected abnormalities in the muscle activity patients displayed during treadmill walking. Abnormally high levels of activity were found in the affected BF and VL and in the unaffected GM and BF. For these muscles, a larger Lokomat induced reduction in activity was found for patients compared to healthy walkers. Conversely, for affected MG^SS^, Lokomat-induced reductions in muscle activity were significantly larger in healthy walkers compared to patients, as activity in patients on the treadmill was significantly lower than in healthy walkers. Nonetheless, these findings seem to suggest that Lokomat guided walking induced reductions irrespective of the nature of abnormalities (e.g. abnormally high or low amplitude) observed during regular treadmill walking. In sum, for some muscles (i.e. affected BF and VL and in the unaffected GM and BF), the present results confirm findings by Coenen et al. [23] showing that patterns of muscle activity during Lokomat guided walking are more similar for stroke patients and healthy walkers. However, the results also indicate that this is mainly due to a general reduction of activity, resulting in abnormally low levels of muscle activity, in both groups.

It is interesting to note that the abnormally high activity that patients displayed during treadmill walking may be related to an adaptive strategy. More specifically, the increased and prolonged activity that was observed in the upper leg muscles (i.e. BF and VL), may indicate prolonged co-activation of these muscles (see [3, 20–22] for similar results). This may be related to a compensatory strategy that serves to provide additional support during stance [20, 21, 43], a strategy that has also been observed in toddlers [44], patients with diabetic neuropathy [45], and patients with spinal cord injury [46]. As the Lokomat exoskeleton stabilizes the stance leg, such compensatory co-activation of muscles may no longer be necessary or effective. As a consequence, the abnormally high activity in the affected and unaffected upper leg muscles during treadmill walking was reduced to levels comparable to those of healthy walkers when walking in the Lokomat.

### Hemiparetic gait is more symmetrical during Lokomat guided walking

During treadmill walking, patients showed a significantly shorter relative SS duration on the affected side compared to the unaffected side, resulting in temporal step asymmetry, as has previously been shown by others [3–5]. These asymmetries predominantly stem from impaired single limb support, due to balance deficiencies and difficulty in moving the body over an unstable limb [2], urging patients to prematurely terminate this phase of the gait cycle. During Lokomat guided walking, temporal asymmetry was significantly reduced due to a prolongation of the relative SS duration in the affected limb. The increased temporal symmetry during Lokomat guided walking may be due to the exoskeleton enforcing a predefined, symmetrical stepping pattern. Previous research on healthy walkers has shown that during Lokomat guided walking the modulations of SS durations normally associated with changes in gait speed, are absent [17], suggesting that functional control of step phase durations is partly overruled by exoskeleton guidance. In addition, the stabilization of the stance leg in the Lokomat exoskeleton and imposed guidance may help to overcome impaired single limb support of the affected leg, allowing patients to walk more symmetrically.

### Clinical considerations

The physical support of limb movements to attain safe and successful stepping in the light of impaired control, represents a general principle of locomotor training that is implemented in both manually assisted physical therapy and RAGT. However, motor learning requires active participation of the patient and an inevitable consequence of providing support is that it reduces the need for active contributions. Previous studies on the training of novel hand and arm movements have shown that fully guided movements that do not require active involvement are generally ineffective in increasing task performance [14, 15, 47]. This may be partly due to a mismatch between the task dynamics required during guided movements and those that are involved in the active production of the movement to be learned [48]. Arguably, the potential of the Lokomat as a gait-training device may depend on the extent to which it encourages active contributions from the patient. The present results show that when guidance levels are set to 50%, levels of muscle activity are reduced quite dramatically. Although some muscles still display clearly phased activation profiles during Lokomat guided gait (e.g. unaffected GM, TA), for other muscles these conditions result in an almost complete abolishment of activity that was already low during treadmill walking (affected GM, affected MG). These strong reductions in activity may not represent a favourable condition for locomotor (re-) learning, and may limit long term effects on the rehabilitation outcome. Possible strategies to overcome these limitations and promote neuromuscular activity may entail encouragement or motivational feedback [36, 40, 49, 50] or increasing gait speed [17, 51, 52].

It has been suggested that the ability to develop compensatory mechanisms represents an important mechanism for functional recovery [53]. The present results suggest that Lokomat guided walking may discourage the need for such compensatory strategies, as indicated by the reductions in abnormal muscle activity (VL and BF) and temporal asymmetry in the patient group. To allow exploration of compensatory mechanisms during Lokomat guided walking, exoskeletons should operate in a mode that can selectively target specific impairments in locomotor task performance (e.g. impaired single limb support), following a so-called ‘assist as needed’ paradigm [10–12, 51]. In this paradigm, patients are free to explore (compensatory) patterns, but are assisted when failing to successfully complete the gait cycle. Although the impedance controller that drives the here used type of the Lokomat allows control of the overall guidance level, the timing of movements is fixed [10, 54] and guidance is provided equally throughout the gait cycle. However, recently a ‘path control algorithm’ was developed, that produces a supportive force for the timing of movements, which can be set separately from the guidance force (see e.g. [12, 54]). This allows more flexibly timed limb trajectories and provide opportunities for compensatory control.

### Limitations

A number of potential limitations should be taken into account when considering the present results. First, no instructions were given to participants on whether exoskeleton movements had to be actively ‘reproduced’ or followed more or less passively, although such instructions are known to affect the level of muscular involvement in Lokomat guided gait [36, 40]. Second, foot support was provided by means of elastic foot lifters, which may explain the observed decreased TA activity during swing phase, and reduced MG activity during the stance phase. Foot support during the swing phase will unload the TA, while the restriction of plantar flexion in stance will limit MG activity during push-off. Indeed, the use of foot lifters has been shown to reduce ankle dorsi- and plantar flexor activity [16, 23]. However, it must be noted that the use of foot lifters is a commonly used strategy to prevent unwanted plantar flexion, and concomitant stumbling, during Lokomat guided walking. Third, EMG was only measured during a single session. As such, the patterns recorded may not be representative for multi-session therapy, as especially in novice walkers neuromuscular responses to the Lokomat training environment may change due to habituation or learning effects. Fourth, although the use of FAC scores for inclusion resulted in a group of patients that is representative for the patients targeted for Lokomat guided training, no information was available about specific motor problems in patients (such as balance problems), possibly limiting the interpretation of the observed abnormalities of patients in the context of specific (loco-)motor problems. Finally, we choose to keep guidance, BWS, and treadmill speed constant throughout the experiment, as they are known to affect muscle activity [17]. However, during training, these parameters and their mutual interactions may be effectively exploited to alter gait demands and its underlying neuromuscular control.

## Conclusions

Compared to treadmill walking, Lokomat guided walking was associated with reductions in the amplitude of muscle activity in both stroke patients and healthy walkers, and a reduction in temporal step asymmetry in stroke patients. In patients, the reductions in muscle output were apparent irrespective of whether muscle activity was abnormally high or abnormally low during treadmill walking. The a-specific reductions in muscle activity in Lokomat guided gait should be taken into account when considering the clinical potential of this training environment.

## Additional file

Additional file 1: Complete overview of descriptive data and statistical results. The additional file contains a complete overview of descriptive data and results of all statistical testing in three tables: **Table S1.** Descriptive data and statistical results of EMG patterns of leg muscles of the affected limb of stroke patients versus the right limb of healthy controls. **Table S2.** Descriptive data and statistical results of EMG patterns of leg muscles of the unaffected limb of stroke patients versus the right limb of healthy controls. **Table S3.** Descriptive data and statistical results of step phase durations of the affected versus the unaffected limb of stroke patients. (DOCX 30 kb)

## Abbreviations

AFO: Ankle Foot Orthoses
BF: Biceps Femoris
BWS: Bodyweight Support
DS1: First double support
DS2: Second double support
EMG: Electromyography
GM: Gluteus Medius
L/R: Left/Right
METc UMCG: Medical Ethical Committee of the University Medical Center Groningen
MG: Medial Gastrocnemius
RAGT: Robot Assisted Gait Training
SD: Standard deviation
SS: Single support
SW: Swing
TA: Tibialis Anterior
VL: Vastus Lateralis

## References

[1] Schmidt H, Volkmar M, Werner C, Helmich I, Piorko F, Krüger, J, Hesse S. Muscle activation patterns of healthy subjects during floor walking and stair climbing on an end-effector-based gait rehabilitation robot. Int Conf Rehabil Robot. 2007;2007:1077–084.

[2] Von Schroeder HP, Coutts RD, Lyden PD, Billings E (1995). Gait parameters following stroke: a practical assessment. J Rehabil Res Dev.

[3] Olney SJ, Richards C (1996). Hemiparetic gait following stroke. Part 1: Characteristics. Gait Posture.

[4] Wall JC, Turnbull GI (1986). Gait asymmetries in residual hemiplegia. Arch Phys Med Rehabil.

[5] Chen G, Patten C, Kothari DH, Zajac FE (2005). Gait differences between individuals with post-stroke hemiparesis and non-disabled controls at matched speeds. Gait Posture.

[6] Titianova EB, Tarkka IM (1995). Asymmetry in walking performance and postural sway in patients with chronic unilateral cerebral infarction. J Rehabil Res Dev.

[7] Mehrholz J, Elsner B, Werner C, Kugler J, Pohl M. Electromechanical‐assisted training for walking after stroke. Cochrane Libr. 2013;CD006185. 10.1002/14651858.CD006185.pub3 PMC6465057 23888479

[8] Veerbeek JM, van Wegen E, van Peppen R, van der Wees PJ, Hendriks E, Rietberg M, Kwakkel G (2014). (2014). What is the evidence for physical therapy poststroke? A systematic review and meta-analysis. PLoS One.

[9] Colombo G, Joerg M, Schreier R, Dietz V (2000). Treadmill training of paraplegic patients using a robotic orthosis. J Rehabil Res Dev.

[10] Duschau-Wicke A, von Zitzewitz J, Caprez A, Lunenburger L, Riener R (2010). Path control: a method for patient-cooperative robot-aided gait rehabilitation. Neural Syst Rehab Eng, IEEE Trans.

[11] Riener R, Lünenburger L, Jezernik S, Anderschitz M, Colombo G, Dietz V (2005). Patient-cooperative strategies for robot-aided treadmill training: first experimental results. Neural Syst Rehab Eng, IEEE Trans.

[12] Riener R, Lünenburger L, Maier I, Colombo G, Dietz EV (2010). Locomotor Training in Subjects with Sensori-Motor Deficits: An Overview of the Robotic Gait Orthosis Lokomat. J Healthc Eng.

[13] Dobkin BH (2004). Neurobiology of rehabilitation. Ann N Y Acad Sci.

[14] Lotze M (2003). Motor learning elicited by voluntary drive. Brain.

[15] Kaelin-Lang A, Sawaki L, Cohen LG (2005). Role of voluntary drive in encoding an elementary motor memory. J Neurophysiol.

[16] Hidler JM, Wall AE (2005). Alterations in muscle activation patterns during robotic-assisted walking. Clin Biomech.

[17] van Kammen K, Boonstra AM, van der Woude LH, Reinders-Messelink HA, den Otter R (2016). The combined effects of guidance force, bodyweight support and gait speed on muscle activity during able-bodied walking in the Lokomat. Clin Biomech.

[18] Dietz V, Berger W (1984). Interlimb coordination of posture in patients with spastic paresis. Brain.

[19] Lamontagne A, Richards CL, Malouin F (2000). Coactivation during gait as an adaptive behavior after stroke. J Electromyogr Kinesiol.

[20] Den Otter AR, Geurts ACH, Mulder T, Duysens J (2006). (2006). Gait recovery is not associated with changes in temporal patterning of muscle activity during treadmill walking in patients with post-stroke hemiparesis. Clin Neurophysiol.

[21] Den Otter AR, Geurts ACH, Mulder T, Duysen J (2007). Abnormalities in the temporal patterning of lower extremity muscle activity in hemiparetic gait. Gait Posture.

[22] Buurke JH, Nene AV, Kwakkel G, Erren-Wolters V, IJzerman MJ, Hermens HJ (2008). (2008). Recovery of gait after stroke: what changes?. Neuro Rehabil Neural Repair.

[23] Coenen P, van Werven G, van Nunen MP, Van Dieen JH, Gerrits KH, Janssen TWJ (2012). Robot-assisted walking vs overground walking in stroke patients: an evaluation of muscle activity. J Rehabil Med.

[24] Ivanenko YP, Grasso R, Macellari V, Lacquaniti F (2002). Control of foot trajectory in human locomotion: role of ground contact forces in simulated reduced gravity. J Neurophysiol.

[25] Finch L, Barbeau H, Arsenault B (1991). Influence of body weight support on normal human gait: development of a gait retraining strategy. Phys Ther.

[26] Hesse S, Helm B, Krajnik J, Gregoric M, Mauritz KH (1997). Treadmill training with partial body weight support: influence of body weight release on the gait of hemiparetic patients. NeuroRehabil Neural Repair.

[27] Holden MK, Gill KM, Magliozzi MR, Nathan J, Piehl-Baker L (1984). Clinical gait assessment in the neurologically impaired. Reliability and meaningfulness. Phys Ther.

[28] Cockrell JR, Folstein MF (2002). Mini-mental state examination. Principles and practice of geriatric psychiatry.

[29] World Medical Association declaration of Helsinki: Ethical principles for medical research involving human subjects. JAMA. 2013;310(20):2191–194. 10.1001/jama.2013.281053 24141714

[30] Hof AL, Elzinga H, Grimmius W, Halbertsma JPK (2002). Speed dependence of averaged EMG profiles in walking. Gait Posture.

[31] Den Otter AR, Geurts ACH, Mulder T, Duysens J (2004). Speed related changes in muscle activity from normal to very slow walking speeds. Gait Posture.

[32] Hussain S, Xie SQ, Liu G (2011). Robot assisted treadmill training: mechanisms and training strategies. Med Eng Phys.

[33] Õunpuu S, Winter DA (1989). Bilateral electromyographical analysis of the lower limbs during walking in normal adults. Electroencephalogr Clin Neurophysiol.

[34] Freriks B, Hermens H, Disselhorst-Klug C, Rau G, Hermens H (1999). The recommendations for sensor and sensor placement procedures for surface electromyography. European recommendations for surface electromyography.

[35] Hermens H (1999). European recommendations for surface electromyography.

[36] Schuler TA, Müller R, Van Hedel HJ (2013). Leg surface electromyography patterns in children with neuro-orthopedic disorders walking on a treadmill unassisted and assisted by a robot with and without encouragement. J of Neuroeng and Rehabil.

[37] Bourbonnais D, Noven S (1989). Weakness in patients with hemiparesis. Am J Occup Ther.

[38] Neckel N, Pelliccio M, Nichols D, Hidler J (2006). Quantification of functional weakness and abnormal synergy patterns in the lower limb of individuals with chronic stroke. J Neuroeng Rehabil.

[39] Benjamini Y, Hochberg Y. Controlling the false discovery rate: a practical and powerful approach to multiple testing. J R Stat Soc Series B Stat Methodol. 1995;57(1):289–300.

[40] Israel JF, Campbell DD, Kahn JH, Hornby TG (2006). Metabolic costs and muscle activity patterns during robotic-and therapist-assisted treadmill walking in individuals with incomplete spinal cord injury. Phys Ther.

[41] Krewer C, Müller F, Husemann B, Heller S, Quintern J, Koenig E (2007). The influence of different Lokomat walking conditions on the energy expenditure of hemiparetic patients and healthy subjects. Gait Posture.

[42] van Nunen MP, de Haan A (2012). Exercise intensity of robot-assisted walking versus overground walking in nonambulatory stroke patients. J Rehabil Res Dev.

[43] Higginson JS, Zajac FE, Neptune RR, Kautz SA, Delp SL (2006). Muscle contributions to support during gait in an individual with post-stroke hemiparesis. J Biomech.

[44] Okamoto T, Okamoto K, Andrew PD (2003). Electromyographic developmental changes in one individual from newborn stepping to mature walking. Gait Posture.

[45] Kwon OY, Minor SD, Maluf KS, Mueller MJ (2003). Comparison of muscle activity during walking in subjects with and without diabetic neuropathy. Gait Posture.

[46] Leroux A, Fung J, Barbeau H (1999). Adaptation of the walking pattern to uphill walking in normal and spinal-cord injured subjects. Exp Brain Res.

[47] Liu J, Cramer SC, Reinkensmeyer DJ (2006). Learning to perform a new movement with robotic assistance: comparison of haptic guidance and visual demonstration. J Neuroeng Rehabil.

[48] Reinkensmeyer DJ, Patton JL (2009). Can robots help the learning of skilled actions?. Exerc Sport Sci Rev.

[49] Mazzoleni S, Boldrini E, Laschi C, Carrozza MC, Stampacchia G, Rossi B. Changes on EMG activation in healthy subjects and incomplete SCI patientsfollowing a robot-assisted locomotor training. Int Conf Rehabil Robot. 2011;2011:1–6. 10.1109/ICORR.2011.5975467 22275665

[50] Marchal-Crespo L, Reinkensmeyer DJ. Review of control strategies for robotic movement training after neurologic injury. J Neuroeng Rehabil. 2009;6(1)1. 10.1186/1743-0003-6-20 PMC2710333 19531254

[51] Koenig A, Omlin X, Bergmann J, Zimmerli L, Bolliger M, Müller F, Riener R (2011). Controlling patient participation during robot-assisted gait training. J Neuroeng Rehabil.

[52] Van Kammen K, Boonstra A, Reinders-Messelink H, den Otter R (2014). 2014. The combined effects of body weight support and gait speed on gait related muscle activity: a comparison between walking in the Lokomat exoskeleton and regular treadmill walking. PLoS One.

[53] Kwakkel G, Kollen B, Lindeman E (2004). Understanding the pattern of functional recovery after stroke: facts and theories. Restor Neurol Neurosci.

[54] Duschau-Wicke A, Caprez A, Riener R (2010). Patient-cooperative control increases active participation of individuals with SCI during robot-aided gait training. J of Neuroeng and Rehabil.

