# Supplementary material for: Differences in muscle activity and temporal step parameters between Lokomat guided walking and treadmill walking in post-stroke hemiparetic patients and healthy walkers
Source: J Neuroeng Rehabil. 2017 Apr 20;14:32. doi: 10.1186/s12984-017-0244-z (PMC5397709; doi:10.1186/s12984-017-0244-z)
Supplement: Additional file 1: — Complete overview of descriptive data and statistical results. The additional file contains a complete overview of descriptive data and results of all statistical testing in three tables: Table S1. Descriptive data and statistical results of EMG patterns of leg muscles of the affected limb of stroke patients versus the right limb of healthy controls. Table S2. Descriptive data and statistical results of EMG patterns of leg muscles of the unaffected limb of stroke patients versus the right limb of healthy controls. Table S3. Descriptive data and statistical results of step phase durations of the affected versus the unaffected limb of stroke patients. (DOCX 30 kb) [file 12984_2017_244_MOESM1_ESM.docx]

**Table S1. Descriptive data and statistical results of EMG patterns of leg muscles of the affected limb of stroke patients versus the right limb of healthy controls,** during the 4 phases of gait. Mean activity and associated standard deviations (μV) during both conditions. Test results of independent t-test comparison of EMG patterns of stroke patients and healthy controls during treadmill walking and univariate results of the Repeated Measure ANOVA on the main and interaction effects of Group (stroke vs controls) and Condition (treadmill vs Lokomat).

|  | Treadmill | | Lokomat | | T – test | ANOVA | | |
| --- | --- | --- | --- | --- | --- | --- | --- | --- |
|  | Stroke | Controls | Stroke | Controls |  | Group | Condition | Condition*Group |
|  | *Mean (SD)* | *Mean (SD)* | *Mean (SD)* | *Mean (SD)* | t (18) | F (1,18) | F(1,18) | F(1,18) |
| Gluteus medius | | | | | | | | |
| DS1^a^ | 18.8 (15.1) | 36.0 (21.2) | 8.3 (4.6) | 19.3 (9.0) | -2.09 | **10.07**** | **9.76**** | 0.49 |
| SS^b^ | 9.6 (5.9) | 26.2 (14.0) | 5.2 (2.5) | 11.5 (8.4) | **-3.49**** | **18.61***** | **11.30**** | 3.31 |
| DS2^c^ | 12.2 (12.0) | 5.0 (1.6) | 7.4 (5.9) | 6.6 (3.3) | 1.89 | 2.09 | 1.45 | 5.56 |
| SW^d^ | 9.4 (6.8) | 5.9 (2.3) | 5.8 (3.4) | 7.7 (4.2) | 1.52 | 0.24 | 0.63 | 5.55 |
| Biceps Femoris | | | | | | | | |
| DS1^a^ | 51.2 (31.4) | 26.7 (14.2) | 28.7 (18.0) | 26.6 (19.3) | **2.25*** | 3.04 | 3.63 | 3.51 |
| SS^b^ | 24.9 (9.8) | 9.2 (5.9) | 15.1 (8.5) | 13.6 (10.6) | **4.36***** | 7.60 | 1.21 | **8.59**** |
| DS2^c^ | 14.5 (9.9) | 4.7 (2.9) | 10.3 (7.5) | 10.8 (11.2) | **3.01*** | 2.21 | 0.19 | 5.63 |
| SW^d^ | 21.7 (11.9) | 17.9 (7.7) | 18.9 (12.3) | 14.6 (5.8) | 0.85 | 1.42 | 1.22 | 0.01 |
| Vastus Lateralis | | | | | | | | |
| DS1^a^ | 79.1 (54.7) | 44.3 (33.7) | 19.1 (15.5) | 32.1 (24.0) | 1.71 | 0.91 | **11.19**** | **4.89*** |
| SS^b^ | 39.0 (20.8) | 17.2 (12.5) | 12.6 (7.3) | 18.2 (12.0) | **2.84*** | 3.15 | **8.67**** | **10.06**** |
| DS2^c^ | 21.5 (8.3) | 5.7 (3.4) | 10.7 (7.5) | 7.7 (3.3) | **5.54***** | **28.33***** | 4.55 | **9.48**** |
| SW^d^ | 20.8 (10.6) | 7.3 (3.4) | 9.9 (6.1) | 8.6 (3.4) | **3.83**** | **15.73**** | 4.39 | **7.22*** |
| Medial Gastrocnemius | | | | | | | | |
| DS1^a^ | 23.5 (16.5) | 14.6 (18.0) | 17.5 (15.6) | 18.4 (31.3) | 1.16 | 0.27 | 0.04 | 0.80 |
| SS^b^ | 20.2 (19.1) | 89.0 (50.0) | 13.9 (13.1) | 26.2 (19.5) | **-4.07**** | **13.12**** | **26.52***** | **17.76**** |
| DS2^c^ | 12.8 (14.7) | 25.0 (18.2) | 11.3 (11.4) | 31.8 (29.8) | -1.64 | 4.29 | 0.43 | 1.09 |
| SW^d^ | 12.8 (9.6) | 8.4 (6.6) | 12.7 (10.4) | 15.1 (11.5) | 1.21 | 0.07 | 4.76 | 5.15 |
| Tibialis Anterios | | | | | | | | |
| DS1^a^ | 35.6 (29.0) | 75.1 (33.9) | 22.5 (37.1) | 63.6 (27.0) | **-2.79*** | **10.99**** | 2.66 | 0.01 |
| SS^b^ | 22.8 (13.4) | 16.1 (8.2) | 13.0 (15.5) | 18.1 (19.9) | 1.35 | 0.02 | 1.53 | 3.59 |
| DS2^c^ | 38.9 (34.1) | 21.8 (12.4) | 14.3 (16.3) | 22.1 (23.5) | 1.49 | 0.26 | **6.06**** | 6.30 |
| SW^d^ | 41.2 (36.3) | 54.8 (22.7) | 19.5 (27.8) | 31.0 (23.2) | -1.01 | 1.21 | **19.78***** | 0.05 |

^a^ first double support phase; ^b^ single support phase; ^c^ second double support phase; ^d^ swing phase; **bold = significant: * < 0.05; ** < 0.01; *** < 0.001**

**Table S2. Descriptive data and statistical results of EMG patterns of leg muscles of the unaffected limb of stroke patienst versus the right limb of healthy controls,** during the 4 phases of gait. Mean activity and associated standard deviations (μV) during both conditions. Test results of independent t-test comparison of EMG patterns of stroke patients and healthy controls during treadmill walking and univariate results of the Repeated Measure ANOVA on the main and interaction effects of Group (stroke vs controls) and Condition (treadmill vs Lokomat).

|  | Treadmill | | Lokomat | | T – test | ANOVA | | |
| --- | --- | --- | --- | --- | --- | --- | --- | --- |
|  | Stroke | Controls | Stroke | Controls |  | Group | Condition | Condition*Group |
|  | *Mean (SD)* | *Mean (SD)* | *Mean (SD)* | *Mean (SD)* | t (18) | F (1,18) | F(1,18) | F(1,18) |
| Gluteus medius | | | | | | | | |
| DS1^a^ | 52.6 (34.3) | 36.0 (21.2) | 30.9 (24.4) | 19.3 (9.0) | 1.30 | 2.22 | **14.32**** | 0.25 |
| SS^b^ | 32.6 (23.8) | 26.2 (13.9) | 21.0 (22.2) | 11.5 (8.4) | 0.74 | 1.20 | **13.54**** | 0.19 |
| DS2^c^ | 10.4 (7.0) | 5.0 (1.6) | 6.9 (5.6) | 6.6 (3.3) | 2.38 | 2.86 | 0.50 | 3.39 |
| SW^d^ | 13.1 (7.1) | 5.9 (2.3) | 7.6 (5.1) | 7.7 (4.2) | **3.09*** | 3.62 | 2.63 | **9.87**** |
| Biceps Femoris | | | | | | | | |
| DS1^a^ | 61.2 (42.1) | 26.7 (14.9) | 35.3 (24.2) | 26.6 (19.3) | **2.46*** | 4.12 | 5.23 | 5.08 |
| SS^b^ | 38.9 (21.5) | 9.2 (5.9) | 20.3 (12.1) | 13.6 (10.6) | **4.23**** | **11.07**** | 6.54 | **17.36**** |
| DS2^c^ | 14.0 (8.2) | 4.7 (2.9) | 12.9 (9.7) | 10.8 (11.2) | **3.36**** | 3.00 | 1.57 | 3.21 |
| SW^d^ | 30.1 (29.0) | 17.9 (7.7) | 22.6 (21.5) | 14.6 (5.8) | 1.29 | 1.63 | 4.02 | 0.61 |
| Vastus Lateralis | | | | | | | | |
| DS1^a^ | 63.2 (34.9) | 44.3 (33.7) | 21.8 (16.0) | 32.1 (24.0) | 1.23 | 0.16 | **15.25**** | 4.50 |
| SS^b^ | 35.6 (20.6) | 17.2 (12.5) | 17.3 (14.6) | 18.2 (12.0) | 2.41 | 2.41 | 4.90 | 6.07 |
| DS2^c^ | 10.9 (6.9) | 5.7 (3.4) | 12.7 (11.7) | 7.7 (3.3) | 2.14 | 3.77 | 1.06 | 0.00 |
| SW^d^ | 19.2 (14.3) | 7.3 (3.4) | 9.6 (6.0) | 8.6 (3.4) | 2.58 | 5.20 | 3.36 | 5.92 |
| Medial Gastrocnemius | | | | | | | | |
| DS1^a^ | 19.7 (15.6) | 14.6 (18.0) | 17.1 (14.4) | 18.4 (31.3) | 0.68 | 0.06 | 0.02 | 0.38 |
| SS^b^ | 38.3 (32.1) | 89.0 (50.0) | 16.1 (16.2) | 26.2 (19.5) | -2.70 | 5.97 | **34.01***** | **7.74*** |
| DS2^c^ | 15.6 (11.3) | 25.0 (18.2) | 14.2 (13.8) | 31.8 (29.8) | -1.39 | 3.26 | 0.35 | 0.77 |
| SW^d^ | 12.4 (8.6) | 8.4 (6.6) | 16.6 (17.9) | 15.1 (11.5) | 1.18 | 0.36 | 4.37 | 0.24 |
| Tibialis Anterios | | | | | | | | |
| DS1^a^ | 83.5 (40.5) | 75.1 (33.9) | 43.9 (29.4) | 63.6 (27.0) | 0.50 | 0.26 | **6.75*** | 2.04 |
| SS^b^ | 37.0 (20.5) | 16.1 (8.2) | 22.0 (20.1) | 18.1 (19.9) | **3.00*** | 5.05 | 1.24 | 2.16 |
| DS2^c^ | 34.6 (13.3) | 21.8 (12.4) | 16.3 (10.4) | 22.1 (23.5) | 2.22 | 0.42 | 4.07 | 4.29 |
| SW^d^ | 77.5 (33.0) | 54.8 (22.7) | 25.4 (18.6) | 31.0 (23.2) | 1.79 | 0.95 | **28.44***** | 4.21 |

^a^ first double support phase; ^b^ single support phase; ^c^ second double support phase; ^d^ swing phase; **bold = significant: * < 0.05; ** < 0.01; *** < 0.001**

**Table S3. Descriptive data and statistical results of step phase durations of the affected versus the unaffected limb of stroke patients,** during two gait phases. Mean activity and associated standard deviations (% gait cycle) during both conditions. Univariate results of the Repeated Measure ANOVA on the main and interaction effects of Limb (affected versus unaffected) and Condition (treadmill vs Lokomat).

|  | Treadmill | | Lokomat | |  | ANOVA |  |
| --- | --- | --- | --- | --- | --- | --- | --- |
|  | Affected | Unaffected | Affected | Unaffected | Limb | Condition | Condition*Limb |
|  | *Mean (SD)* | *Mean (SD)* | *Mean (SD)* | *Mean (SD)* | F(1,9) | F(1,9) | F(1,9) |
| First Double Support Phase | 11.3 (4.6) | 12.7 (3.1) | 6.8 (3.0) | 7.1 (2.9) | 1.36 | **17.47**** | 0.34 |
| Single Support Phase | 30.4 (4.8) | 45.6 (4.1) | 40.9 (4.3) | 45.2 (3.6) | **36.06***** | **17.47**** | **32.18***** |

**bold = significant: * < 0.05; ** < 0.01; *** < 0.001**
